# Effect of comorbid ADHD on mortality in women with borderline personality disorder

**DOI:** 10.1186/s40479-022-00196-8

**Published:** 2022-11-01

**Authors:** Efthymios Kouppis, Bengt Gerdin, Charlotte Björkenstam, Emma Björkenstam, Lisa Ekselius

**Affiliations:** 1 Department of Medical Sciences, Uppsala University, Uppsala, Sweden; 2 Department of Surgical Sciences, Uppsala University, Uppsala, Sweden; 3 Department of Clinical Neuroscience, Karolinska Institutet, Stockholm, Sweden; 4 Department of Women’s and Children’s Health, Uppsala University, Akademiska Sjukhuset, 751 85 Uppsala, Sweden

**Keywords:** Attention deficiency and hyperactivity disorder, Borderline personality disorder, Clinical diagnosis, Comorbidity, Mortality, Register, Women

## Abstract

**Background:**

Many similarities exist between borderline personality disorder (BPD) and attention-deficit/hyperactivity disorder (ADHD), more so in women than in men. People with comorbid ADHD and BPD represent a subgroup of BPD patients with distinct symptom expression and, consequently, a different prognosis. We used Swedish national high quality registers to assess whether such comorbidity is related to increased mortality risk. The study focused on women with BPD because they are more likely than men to be clinically diagnosed with BPD and present a higher mortality risk, especially for unnatural causes of death, including suicide.

**Findings:**

In a cohort of 15 847 women diagnosed with BPD a subsequent clinical diagnosis of ADHD did not influence the overall risk of mortality, including suicide.

**Conclusions:**

Women with comorbid ADHD and BPD have a similar mortality risk as those only diagnosed with BPD.

## Introduction

Several symptoms of borderline personality disorder (BPD) overlap with those of attention-deficit/hyperactivity disorder (ADHD), which makes differential diagnosis difficult [1]. Subgroups of patients exhibit common symptomatology, particularly for emotional dysregulation [2], which may reflect differences in clinical problems and prognosis. In a clinical setting it is common that patients initially diagnosed with BPD are perceived to have (or develop) a clinical picture suggesting ADHD comorbidity [3, 4].

DSM-5 describes BPD based on the presence of a pervasive pattern of unstable interpersonal relationships, self-image and affects, as well as marked impulsivity present in diverse contexts [5]. In ICD-10 BPD corresponds to emotionally unstable personality disorder (EUPD) with similar symptomatology [6]. The DSM-5 and the EUPD descriptions denote wide qualitative and quantitative variations in symptomatology. Two key features in the two classification systems are self-harm and suicidal behaviour. There has been considerable search for individual characteristics linked to the increased risk of suicide in this patient group [7–9].

ADHD, characterized by inattention, hyperactivity, and impulsivity, i.e., symptoms that may also lead to a diagnosis of BPD, is a lifespan disorder with roots in early childhood and branches extending into adolescence and adulthood [10]. Consequences in many aspects of life such as inability to give close attention to details or make careless mistakes in schoolwork, at work, or with other activities, are actually required for a diagnosis (see e.g. [10]).

The main common symptom of BPD and ADHD is impulsivity [1, 4]. Emotional dysregulation, or impulsiveness, is a central component of ADHD [11] and a critical dimension in BPD [12]. Yet, there are differences between BPD and ADHD [1, 13]. For instance, symptoms of self-destruction and affect dysregulation appear to be more severe in patients with BPD [14]. Both conditions have an increased mortality risk, although more pronounced in BPD, mainly driven by death from unnatural causes, e.g., accident or suicide [15–18].

Less is known on whether patients diagnosed with BPD and who fulfil criteria for ADHD belong to a subgroup with higher mortality risk. Such comorbidity signals a more severe clinical presentation [1, 19], but this does not as such imply a higher mortality risk. In the present study we provide information about the effect of comorbid ADHD on the mortality risk in women with BPD. We have focused on women given that they are three times more likely to be diagnosed with clinical BPD than men [17] despite a similar population prevalence in both sexes [20]. Women diagnosed with BPD also have a higher risk of death, especially for unnatural causes of death [17, 21]. Research shows that women with ADHD and BPD share more clinical features than men [14].

## Material and methods

### Study population

The Swedish National Patient Register (NPR) was used to gather information about all women first diagnosed with EUPD/BPD (ICD-10: F60.3) from 2006 to 2015 and aged 18—64 years at the date of diagnosis. The NPR is a population-based register that holds information on inpatient care since 1987 and specialized outpatient care since 2001. The register is of good quality with a high validation rate [22]. Based on the ICD-10 classification system, the NPR is validated for diagnoses of personality disorder. In a previous validation study a virtually complete diagnostic overlap occurred between a diagnosis of EUPD in the NPR and diagnostic criteria for BPD according to DSM-5 [23]. Based on this finding, we have accepted the term BPD. We obtained information about all diagnoses given in inpatient and outpatient care. Causes of death, classified according to ICD-10, were obtained from the Cause of Death Register, which has a high validity; causes of death are missing in only 0.5% of the deceased [24].

### Exposure and outcomes

Exposure was a diagnosis of adult ADHD occurring after the BPD diagnosis (ICD-10: F90). The primary outcomes were all-cause mortality, natural and unnatural causes of death, and suicide (ICD-10: X60-X84 and Y10-Y34).

### Confounders

Education level, obtained from the Participation in Education Register [25], was used as a proxy for socioeconomic status [26]. The analyses were also adjusted for year of birth, age at their first personality disorder diagnosis, parity, somatic and psychiatric comorbidity, and inpatient care as a proxy for severity as this is associated with a higher mortality risk than outpatient care alone in patients with personality disorders [21, 27] (Table 2).

### Statistical analyses

We performed Cox regression to study time to death during follow-up using SAS version 9.2 (SAS Institute Inc., Cary, NC, USA). We assessed person-years at risk by totalling the years that the individuals were alive during follow-up. The entry date was defined as the date of the first diagnosis of personality disorder, even if a specific diagnosis of BPD appeared later; the exit date was defined as the date of death or the end of follow-up (31 December 2015), whichever occurred first. The regressions calculated person-years for comorbid BPD-ADHD from the ADHD diagnosis. This calculation counteracted any bias based on early mortality in persons who would have been classified with ADHD if they had survived long enough. We performed two separate regressions: one adjusted for year of birth and education level, and one with additional adjustment for age at diagnosis, psychiatric and somatic comorbidity, severity, and parity. Results are reported as hazard ratios (HRs) with 95% confidence intervals, CIs.

## Results

Of the 15 847 women diagnosed with BPD, 4 325 (27.3%) were later diagnosed with ADHD (Table 1). Of these 4 325 women, 1 793 (41.5%) were parous and 2 281 (52.7%) received inpatient care. There was no difference in parity or extent of inpatient care between the two groups. Those with comorbid ADHD were slightly younger when diagnosed with BPD.

**Table 1:** Characteristics of the study population

			Number of women			Cause of death		
	Age at diagnosis	Age at death	Total	Alive	Deceased	Natural	All unnatural	Suicide
	M (SD)	M (SD)	N (%)	N (%)	N (%)	n	n	n
All	29.2 (9.9)	37.5 (12.9)	15 847 (100%)	15 491 (100%)	356 (100%)	81	275	237
­Only BPD (100%)	29.8 (10.2)	38.5 (13.3)	11 522 (72.7%)	11 246 (72.6%)	276 (77.5%)	69	207	182
­BPD and ADHD (100%)	27.8 (8.9)	34.1 (11.0)	4 325 (27.3%)^1^	4 245 (27.4%)	80 (22.5%)	12	68	55
Nulliparous			9 559 (100%)	9 337 (100%)	222 (100%)	49	173	155
­Only BPD (44.3%)^2^			7 027 (73.5%)	6 846 (73.3%)	181 (81%)	44	137	126
­BPD and ADHD (58.5%)^3^			2 532 (26.5%)	2 491 (26.7%)	41 (19%)	5	36	29
Parous			6 288 (100%)	6 154 (100%)	134 (100%)	32	102	82
­Only BPD (55.7%)^2^			4 495 (71.5%)	4 400 (71.5%)	95 (71%)	25	70	56
­BPD and ADHD (41.5%)^3^			1 793 (29.5%)	1 754 (28.5%)	39 (29%)	7	32	26
Inpatient care (IC)			7 534 (100%)	7 249 (100%)	285 (100%)	53	232	199
­Only BPD (45.6%)^2^			5 253 (69.7%)	5 036 (69.5%)	217 (76%)	44	173	151
­BPD and ADHD (52.7%)^3^			2 281 (30.3%)	2 213 (30.5%)	68 (24%)	9	59	48
Outpatient care only			8 313 (100%)	8 242 (100%)	71 (100%)	28	43	38
­Only BPD (54.4%)^2^			6 269 (75.4%)	6 210 (75.3%)	59 (83%)	25	34	32
­BPD and ADHD (47.3%)^3^			2 044 (24.6%)	2 032 (24,7%)	12 (17%)	3	9	7

^1^ 1344 of those were registered with an ADHD diagnosis before index and were calculated to belong to the BPD-ADHD group from index; ^2^ of all “Only BPD”, ^3^ of all “BPD and ADHD”

During the follow-up, 356 patients died, representing 2.2% of all women. A detailed description of the study population characteristics, crude numbers, and proportions of deaths partitioned into natural, unnatural, and suicide for the group with ADHD and the one without is presented in Table 1.

Women with BPD and a comorbid ADHD diagnosis had a marginally higher mortality risk for unnatural death than those with BPD alone as assessed in regression models only adjusted for education and year of birth, HR 1.40 (95% CI 1.06–1.85) (Table 2). When assessed in the fully adjusted model, also adjusted for parity, age at their first personality disorder diagnosis, psychiatric and somatic comorbidity, and severity, there was no difference in any of the mortality assessments: for example, HR 1.22 (95% CI 0.92–1.63) for unnatural death and 1.18 (95% CI 0.86–1.61) for suicide.

**Table 2:** Hazard ratios with 95% confidence intervals for all-cause mortality, natural and unnatural deaths, and suicides

	BPD	BPD + ADHD
**All-cause mortality**		
Rate per 100,000 person-years	442.1 (392.9–497.5)	534.2 (429.1–665.1)
Model I^a^	1 (REF)	1.32 (1.02–1.70)
Model II^b^	1 (REF)	1.16 (0.89–1.50)
**Natural death**		
Rate per 100,000 person-years	110.5 (87.3–139.9)	80.13 (45.51–141.1)
Model I^a^	1 (REF)	1.06 (0.57–1.98)
Model II^b^	1 (REF)	0.96 (0.51–1.81)
**Unnatural death**		
Rate per 100,000 person-years	331.6 (289.4–380)	454.1 (358–575.9)
Model I^a^	1 (REF)	1.40 (1.06–1.85)
Model II^b^	1 (REF)	1.22 (0.92–1.63)
**Suicide**		
Rate per 100,000 person-years	291.5(252.1–337.1)	367.3 (282–478.3)
Model I^a^	1 (REF)	1.32 (0.97–1.80)
Model II^b^	1 (REF)	1.18 (0.86–1.61)

^a^ Adjusted for education categorized into three groups: compulsory school (≤ 9 years of education), high school (10–12 years), and college or university (≥ 13 years), and for year of birth

^b^ Adjusted for education, year of birth, age at their first personality disorder diagnosis, parity, somatic and psychiatric comorbidity, and severity. In the Cox regressions somatic diagnoses A00-B99, M00-M99, O00-O79, O85-O99 and S00-T98 were seen as confounders for 30 days, and C00-D48, D50-D89, E00-E90, G00-G99, I00-I99, J00-J99, and K00-K93 for 1 year. Coexisting psychiatric diagnoses F20-F22, F24-F29, F310-F319, and F43.1 were included as lifelong confounders from the first diagnosis, and F10-F19, F100-F107 F23, F30-F39, and F40-F48 were included as confounders for 1 year after each diagnosis

## Discussion

The main finding is that comorbid ADHD has no significant effect on mortality in women previously diagnosed with BPD.

We interpreted inpatient psychiatric care as a sign of severity and observed that the BPD-ADHD group had significantly more inpatient care than the BPD-only group (52.7% vs. 45.6%, X^2^ = 64.44, *p* < 0.001; data not shown). This finding supports a perspective that comorbidity reflects a clinically more severe condition, even if that was not reflected in a higher mortality risk. In contrast, our results suggest that comorbidity represents shared symptoms that are not additive regarding mortality [3].

It is not well understood how the diagnostic similarities between BPD and ADHD are interpreted in routine clinical practice and to what extent comorbidities between BPD and ADHD are identified and registered. In one study 38% of patients with BPD actively screened for ADHD fulfilled requested criteria [3]. This finding suggests a subgroup among patients given a BPD diagnosis alone in routine clinical care who, if adequately diagnosed, would also be given a diagnosis of ADHD. Our study suggests that the mortality in this subgroup would be like that in patients only with BPD. From an overall perspective, the present study does not exclude the possibility that one or more subgroups based on the polythetic criteria set for BPD are negatively affected by comorbidity [3].

The present study requires registers with good coverage, a sufficiently long follow-up and trends in how registered diagnoses are stable over the study period. An ideal situation would have been to have full coverage of in- and outpatient care for the entire cohort from early childhood to death. Such outstanding coverage was not possible. Outpatient care (i.e. the predominant type of care for ADHD) was only partially covered in the registers before the year 2006. Therefore, it was not possible to representatively assess whether there was an early ADHD diagnosis before the index diagnosis for BPD. We had to ignore this limitation and instead focus on the clinical presentation, which had motivated care in real-time.

The results do not answer the extent to which comorbidity as such between ADHD and BPD affects mortality, as neither group is left untreated. Here, persons with diagnosed comorbidity may be more vulnerable and are given more attention during care, including continuous surveillance to maintain and adjust pharmaceutical treatment.

A limitation of the study is that only a subgroup of the background population fulfilling criteria for a diagnosis of BPD receive care and a formal diagnosis and is thereby included in the NPR [23]. Hence, this study cannot be generalized beyond the group that received a clinical diagnosis within health care. Another limitation is related to stability of diagnoses and the concept of comorbidity. The BPD diagnoses are treated as stable entities why all new diagnoses which appear are by definition regarded as comorbid.

## Implications and conclusion

Comorbid ADHD in women with BPD does not negatively affect mortality outcomes.

## Data Availability

According to the Swedish Ethical Review Act, the Data Protection Act, and the Administrative Procedure Act, these data can only be made available after a legal review for researchers who meet the criteria for access to sensitive and confidential data.
